# Whole-Genome Sequencing of 11 High-Risk Clone ST111 Pseudomonas aeruginosa Isolates from French Hospitals

**DOI:** 10.1128/mra.00091-23

**Published:** 2023-06-15

**Authors:** Mikkel Anbo, Mariana Antonova Tavares Chichkova, Yilmaz Emre Gençay, Alex Salazar, Katy Jeannot, Lars Jelsbak

**Affiliations:** a DTU Bioengineering, Technical University of Denmark, Kongens Lyngby, Denmark; b SNIPR Biome, København Ø, Denmark; c Laboratory of Bacteriology, Associated Laboratory to French National Reference Center for Antibiotic Resistance, Teaching Hospital of Besançon, Besançon, France; The University of Arizona

## Abstract

This study reports high-quality genomes of 11 sequence type 111 (ST111) isolates of Pseudomonas aeruginosa. This ST is known for its worldwide dissemination and high capacity to acquire antibiotic resistance mechanisms. This study used long- and short-read sequencing to provide high-quality closed genomes for most of the isolates.

## ANNOUNCEMENT

Pseudomonas aeruginosa is an opportunistic pathogen well known for its problematic role in cystic fibrosis patients and health care-associated infections. Some clones of P. aeruginosa are more prevalent among multiresistant and extensively antibiotic-resistant clinical strains and are known as high-risk clones (HRC) (1). The most frequent HRC are sequence type 235 (ST235), ST111, and ST175 (2).

This study has focused on isolates of the HRC ST111 isolated from 11 French hospitals. These isolates were sequenced in order to expand our knowledge about the interclone type diversity of ST111 and the evolutionary history of this HRC. This data set is particularly interesting because it contains isolates belonging to serotypes O12 and O4. Early evolution of ST111 has been proposed to involve a serotype switch from O4 to O12 prior to its global dissemination (3). This, together with the available genomic resources available for ST111, which shows higher prevalence for serotype O12 (4), reinforces the notion that the ST111 O12 lineage is more successful in health care settings.

Strains were isolated from French hospitals on Mueller-Hinton (MH) medium supplemented with 5% horse blood and 20 mg/L beta-NAD or urine selective medium (urinary tract infection [UTI] chromogenic medium) according to origin (Table 1). Plates were incubated for 16 to 24 h at 35 ± 2°C, and P. aeruginosa isolates were identified by matrix-assisted laser desorption ionization–time of flight (MALDI-TOF) mass spectrometry and morphology according to reference (5). Isolates were stored at −80°C in brain heart medium supplemented with 20% glycerol. Isolates were thawed on LB or MH agar plates and grown in LB broth overnight for 16 to 18 h at 37°C and 200 rpm for DNA extraction. Genomic DNA was extracted using the PureLink genomic DNA minikit (K182001; Thermo Fisher) for Illumina sequencing and the Monarch high-molecular-weight (HMW) DNA extraction kit for tissue (T3060L; New England Biolabs) for Nanopore sequencing. DNA concentration was determined by Qubit and gel electrophoresis. Illumina sequencing was carried out on the NovaSeq 6000 platform with 2- by 150-bp paired-end reads from a library prepared using the Illumina DNA sample prep XT kit. For Nanopore sequencing, the total DNA was barcoded using the rapid barcoding kit (SQK-RBK004) and sequenced on an R9.4.1 flow cell with a MinION device. The reads were base called with MinKNOW (3.1.19).

**TABLE 1:** Strain sequencing and assembly details^*a*^

Strain	Isolation source	No. of reads		Total DNA (Mb)		Nanopore *N*_50_ (kb)	No. of contigs	Completed genome	Assembly size (bp)	GC content (%)	Coverage (×)		Plasmid content and data			Nanopore barcode	Accession no.			
		Illumina	Nanopore	Illumina	Nanopore						Illumina	Nanopore	Plasmid size (bp)	Accession no. for plasmid homologue	ID to and coverage for homologue		BioSample	GenBank	SRA, Illumina	SRA, Nanopore
2856	Tracheal aspirate	6,384,966	6,192	886	210	37.4	2	Yes	7,036,624	65.8	125.8	29.8	24,068	KX912255.1	99.8%, 100%	RB02	SAMN32874240	CP116715.1, CP116716.1	SRR23190728	SRR23191133
2857	Sputum	5,209,304	5,331	720	210	41.8	1	Yes	7,165,834	65.5	100.6	29.3	NA			RB03	SAMN32874241	CP116717.1	SRR23190727	SRR23191132
2858	Urine	4,821,968	4,817	670	210	45.1	2	Yes	6,977,699	65.8	96	30.1	24,299	NA		RB04	SAMN32874242	CP116718.1, CP116719.1	SRR23190725	SRR23191130
2866	Catheter	4,665,392	5,196	669	210	42.8	1	Yes	7,055,952	65.8	94.8	29.8	NA			RB05	SAMN32874243	CP116720.1	SRR23190724	SRR23191129
2867	Bone	5,768,912	5,055	814	210	43.7	1	Yes	7,092,945	65.7	114.8	29.6	NA			RB06	SAMN32874244	CP116721.1	SRR23190723	SRR23191128
2868	Tracheal aspirate	5,048,658	4,400	714	210	48.7	1	Yes	7,262,764	65.7	98.4	28.9	NA			RB07	SAMN32874245	CP116722.1	SRR23190722	SRR23191127
2872	Stool	4,966,380	7,554	709	210	29.2	1	Yes	7,112,365	65.8	99.6	29.5	NA			RB08	SAMN32874246	CP116723.1	SRR23190721	SRR23191126
2875	Rectal screening	5,492,696	16,502	800	210	18.4	3	Yes	7,395,142	65.5	108.2	28.4	245,616	CP041355	98.9%, 56%	RB09	SAMN32874247	CP116727.1, CP116728.1, CP116729.1	SRR23190720	SRR23191125
2879	Urine	4,510,294	6,950	652	86	24.9	9	No	7,333,015	65.7	88.9	11.9	NA			RB10	SAMN32874248	JAQNBM000000000	SRR23190719	SRR23191124
2880	Sputum	6,551,728	5,720	921	210	39.7	1	Yes	7,153,626	65.7	128.8	29.4	NA			RB11	SAMN32874249	CP116724.1	SRR23190718	SRR23191123
2881	Urine	5,700,092	10,906	814	210	26.8	2	Yes	7,200,635	65.7	113.1	29.2	142,661	CP011370.1	100%, 97%	RB12	SAMN32874250	CP116725.1, CP116726.1	SRR23190726	SRR23191131

a Abbreviations: ID, identity; NA, not available.

Trimmomatic 0.39 (6) was used to trim Illumina adapters with settings “LEADING:3 TRAILING:3 SLIDINGWINDOW:4:15 MINLEN:36” and processed with Fastp 0.12.4 (7) for read quality control (QC) (no error correction). The Nanopore reads were demultiplexed, and adapters were trimmed using Porechop 0.2.4 (8) with settings “-barcode_threshold 85.” Duplicated Nanopore reads were removed using SeqKit v2.3.0 (9) and subset to 210 Mb (approximately 30× coverage assuming a genome size of 7 Mb) using Filtlong v0.2.0 (10) with parameters “--min_mean_q 9 -t 210000000.” Unicycler v0.5.0 (11) was used for *de novo* hybrid assembly using default settings. Completeness, rotation, and circularity of assemblies were evaluated and done by Unicycler. Bandage v0.8.1 (12) was used to merge contigs of incomplete assemblies using the “merge all possible nodes” function. Genomes were annotated using the NCBI Prokaryotic Genome Annotation Pipeline v6.4 (13). Assembly statistics are shown in Table 1.

### Data availability.

Detailed information for each strain is listed in Table 1, including GenBank, Sequence Read Archive (SRA), and BioSample accession numbers. The genomic data are publicly available under BioProject no. PRJNA905051. The versions described are the first versions.
